# Growth response and mycoremediation of heavy metals by fungus *Pleurotus* sp*.*

**DOI:** 10.1038/s41598-022-24349-5

**Published:** 2022-11-19

**Authors:** Fereshteh Mohamadhasani, Mehdi Rahimi

**Affiliations:** 1 Department of Biology, Faculty of Science, Payame Noor University (PNU), Tehran, Iran; 2 Department of Biotechnology, Institute of Science and High Technology and Environmental Sciences, Graduate University of Advanced Technology, Kerman, Iran

**Keywords:** Environmental biotechnology, Ecology

## Abstract

Heavy metal contamination (HMs) in water and soil is the most serious problem caused by industrial and mining processes and other human activities. Mycoremediation is a biotechnology that employs fungi to remove toxic contaminants from the environment in an efficient and cost-effective manner. *Pleurotus* spp*.* have been shown to either increase plant growth on metal-contaminated soils by providing more nutrients or by reducing metal toxicity. *Pleurotus* species (J. Lange), a mushroom that can be eaten, has been observed growing on plantations of wood trees in Kerman's orchards. *P.* sp*.* was the subject of this study, which examined the effects of different concentrations of various heavy metals Cobalt (Co), Copper (Cu), and Nickel (Ni) (0, 15, 30, 45, and 60 mg/L) on fungal colony diameters, mycelial dry weights, accumulation of heavy metals, and antioxidative enzymes. The findings revealed that *P.* sp*.* was more tolerant of Co than other metals, so the fungus grew more in the presence of low concentrations of Co and Cu. However, even at concentrations as low as 15 mg/L, Ni greatly inhibited the growth of biomass and colony diameter. Heavy metals increased the activity of superoxide dismutases (SOD) and catalase (CAT) up to 45 mg/L, but an increase in metal concentration above 45 mg/L resulted in a significant decrease in SOD. Metals in mycelium also increased as the concentrations of these heavy metals increased.

## Introduction

Environment contamination with heavy metals has increased over the years. The composition of fungi, plants and microbial activity of the soil can be negatively influenced by the environment contamination due to the processes of mining, smelting, processing and manufacturing of metals and their sub-products^1,2^. Exposure to heavy metals, may be toxic for soil organisms whether of natural origin, such as metalliferous rocks, or of anthropic activity origin such as pollutions, fertilizers, pesticides. The degree of toxicity depends mainly on the metallic elements and their bioavailability in the soil. Various metals, e.g. Zn, Cu, Ni and Mn, are micronutrients but become toxic at higher concentrations. In contrast some metals are not essential for the development of living organisms and are toxic even at very low concentrations e.g. Hg, Cd, Pb^3^.

Macro fungi, are one of the most important micromediators in nature. *Pleurotus* spp. are considered to be the most popular and widely cultivated species worldwide, and this may be due to their low production cost and higher yield*. *This fungi commonly known as the oyster mushroom, belongs to the group of basidiomycete’s fungi. Although approximately 70 species of this genus have been identified. Typical 7 of them are commonly found of mushroom, they are Saprophytic and are mainly found in rainy season especially in forest ecosystem. They are highly adaptive to different climatic condition^4^ and they prefer different agricultural waste for their growth. *Pleurotus* spp. is an important edible mushroom but in recent past their capacity of biosorption has attracted a large number of researchers^5–8^ have indicated that different portions of oyster mushroom differently accumulate heavy metals. *Pleurotus* spp., can be used in bioremediation of the soil contaminated by heavy metals in environment. Mycoremediation is a method of removing heavy metals by fungal biomass, using processes such as degradation, absorption, accumulation and conversion through biological means^9–11^. Biosorption is a passive process than heavy metals get adsorbed on the surface of the biosorbent^12^ exhibiting the tolerance of biosorbent towards heavy metals. The mechanisms like extracellular (chelation and cell wall binding) and intra-cellular (binding to compounds like proteins) sequestration of heavy metals have been proposed as mechanisms for heavy metals tolerance in fungi^13^. Biosorbent from mushrooms can be prepared from mycelium or fruit body (live or dead) and spent mushroom substrate (SMS)^14^. *Pleurotus* spp. of mushrooms (macro-fungi) is more suitable option in terms of heavy metal removal compared to other mushroom species. Fungi have chitin in their walls which can tolerate high concentrations of metals and are capable of growing on medium at low pH and temperature exhibiting excellent mycoremediation potential^15–18^.

The aim of the present study was to assess the tolerance rate of the fungus *Pleurotus* sp*.* grown on pure solid and liquid culture media exposed to different levels of heavy metals, like Cu, Ni, Co and then investigations on the response of the fungus to toxic metals, especially where it is in association with trees, are carrying out here in Kerman, Iran where the fungus grows at sites beneath the trees.

## Materials and methods

### Growing the fungus on solid culture medium

A specific type of fungus that lives on wood trees was collected in Kerman, Iran and was grown on solid Modified Melin Norkrans (MMN) medium^19^ with different concentrations (0, 15, 30, 45, 60 ppm) of each heavy metal sulfate salt copper, nickel and cobalt (Cu^2+^, Ni^2+^, Co^2+^)^20^. The petri dishes were placed at a temperature of 25 °C until the diameter of the colony in the control petri dish (zero concentration of each metal) reached 7 cm. The widest diameter was measured as the radial growth of the mycelia. Then, the diameter of each colony in the widest part was measured with a ruler with an accuracy of 0.1 cm.

### Cultivation of fungal mycelium in MMN liquid culture medium

An equal number of mycelial discs from the sides of mycelia grown in MMN solid medium in sterile conditions were transferred to Erlenmeyer flasks containing liquid medium containing different heavy metals (Co^2+^, Cu^2+^, Ni^2+^) with different concentrations (0, 15, 30, 45, 60 ppm). The flasks were placed in an incubator at 25 °C for 3 weeks so that the mycelium grew in the liquid environment and covered the surface of the liquid. After 21 days, the mycelia were collected, and washed in distilled water, then dried in an oven at 105 °C and finally weighed with a scale with an accuracy of 0.0001.

### Heavy metals tolerance index of fungus

The tolerance index (TI) of a heavy metal is determined for each metal and fungal strain with daily measurements and is obtained by dividing the growth of the fungus exposed to different concentrations of the heavy metal (HMFG) by the growth of the fungus in the control medium (CFG)^21^.

$${\text{TI}} = {\text{HMFG/CFG}}$$

### Mycelium elemental analysis

After drying the mycelium at 105 °C by oven, acid digestion of the mycelium was done using 5 mL of concentrated HNO_3_ and 1 mL of HF for 24 h. Then, the acid digestion solution was used for elemental measurement using Shimadzu AA-670 atomic absorption spectrophotometer^22^.

### Assay of antioxidant enzymes

For SOD activity, the method by Giannopolitis and Ries^18^ that relied on nitroblue tetrazolium photoabsorption (NBT) was used. In it, the extraction solution (50 mM phosphate buffer, pH = 7.8, 0.075 μM NBT, 0.1 mM Na-EDTA, 13 mM armethionine and 50 μL) was mixed with 100 μL of the fungal supernatant, then 75 μM riboflavin was the last component added to the reaction mixture. Then, the tubes were placed under two 15 W fluorescent lamps that the reaction was initiated. After 10 min, by removing the reaction tubes from the light source the reaction was terminated. The reactions with non-illuminated and illuminated served as calibration standards without supernatant. Concerning CAT activity, the modified method by Dhindsa et al.^19^ was used. The volume of solution was 1.5 mL that was made of 100 mmol/L phosphate buffer with pH 7.0, 20 mmol/L H_2_O_2_, 0.1 mmol/L EDTA and 50 μL enzyme extract. The decrease of H_2_O_2_ was monitored at 240 nm and quantified by its molar extinction coefficient (36 mol/L cm) and the results expressed as CAT units/mg of protein^20^.

#### Experimental design and statistical data analysis

The experimental design was completely randomized with 5 treatments, one cultivar and three replications per treatment. Data were analyses by using one-way analysis of variance (ANOVA). Differences between means were considered significant at confidence level of P ≤ 0.05. All statistical analyses were done using the software SPSS package, version 18.0. The Duncan test analysis was done to determine the significant difference between treatments.

## Results

### Response of colony diameter to heavy metals

**Figure 1:**
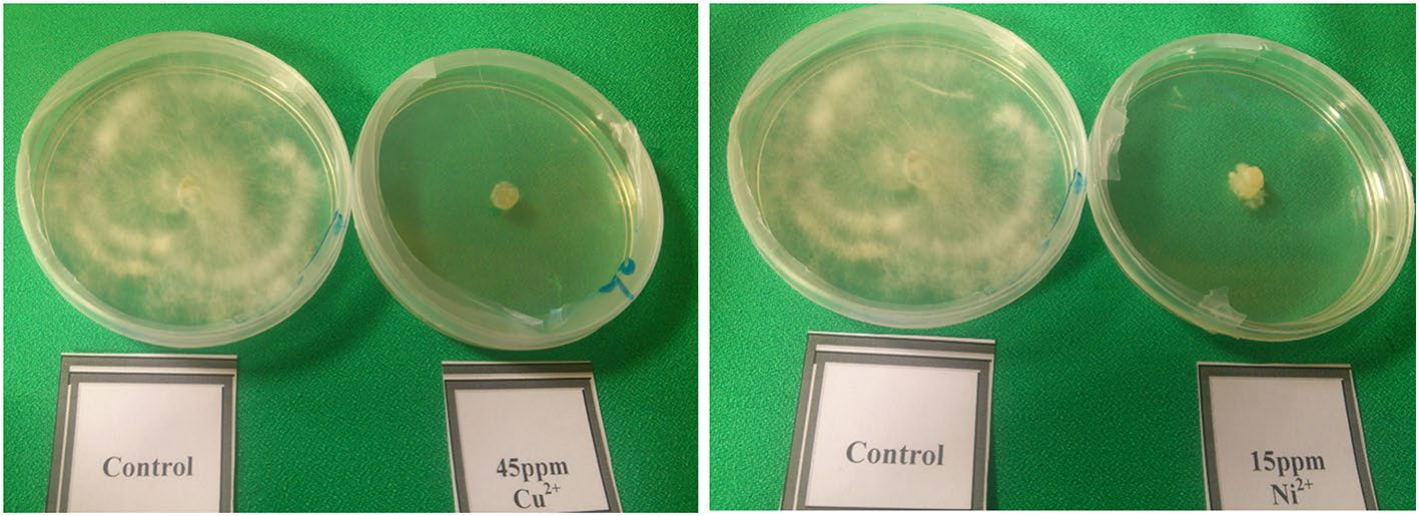
Mycelial growth of mushroom on MMN culture medium.

The responses of *P.* sp*.* to Cobalt, Copper and Nickel as expressed by colony growth on agar plates are shown in Table 1. According to table number 1, *Pleurotus* sp*.* did not grow on medium contain nickel even at concentrations as low as 15 mg/L, therefore, it can be concluded that this fungus is more tolerant to cobalt and copper than nickel (Fig. 1). Results also showed that the diameter of mushroom colonies was significantly decreased with the increasing of metals concentration in the solid culture medium compared to control.

**Table 1:** Comparison of the mean colony diameter of *Pleurotus* sp*.* (cm) grown in MMN solid culture medium containing heavy metals based on Duncan's test.

Metal concentration (mg/L)	Co	Cu	Ni
0	6.5 ± 0.17a	6.9 ± 0.10a	6.85 ± 0.11a
15	2.95 ± 0.024b	2.71 ± 0.05b	0.0 ± 0.00b
30	2.6 ± 0.43b	2.15 ± 0.6b	0.0 ± 0.00b
45	2.1 ± 0.3b	0.0 ± 0.00c	0.0 ± 0.00b
60	0.0 ± 0.00c	0.0 ± 0.00c	0.0 ± 0.00b

Treatments with the same letters are not statistically different.

### Tolerance index

According to the results of Table 2, the reduction of the tolerance index, which varies according to the type and concentration of heavy metal, reflects the inhibitory growth function of heavy metal.

**Table 2:** Tolerance index levels of *Pleurotus* sp*.* in various heavy metals concentrations.

Heavy metals	Concentrations			
	15	30	45	60
Cobalt	0.45	0.4	0.32	0
Copper	0.39	0.31	0	0
Nickel	0	0	0	0

Tolerance index rating values indicate: 0.00–0.39—very low metal tolerance. 0.40–0.59—low metal tolerance. 0.60–0.79—moderate metal tolerance. 0.80–0.99—high metal tolerance. 1.00–> 1.00—very high metal tolerance.

The tolerance rating of *Pleurotus* sp*.* to 15–30 mg/L cobalt concentrations were observed to be low with tolerance indices ranging between 0.4–0.45. However, in copper and nickel in all concentrations indicated very low tolerance revealed by the very low tolerance index values of 0–0.39.

### Measurement of dry weight of mycelium in liquid environment

Examining the results of measuring the dry weight of mycelia in the liquid culture medium showed an inverse relationship between the heavy metal concentration and the dry weight, so that with the increase in the heavy metal concentration in the liquid culture medium, the dry weight was reported to decrease (Table 3).

**Table 3:** Comparison of average dry weight (mg) of *Pleurotus* sp*.* mycelia grown in MMN liquid culture medium containing heavy metals based on Duncan's test.

Metal concentration (mg/L)	Co	Cu	Ni
0	68 ± 0.02a	65 ± 0.02a	68.4 ± 0.02a
15	60 ± 0.01a	58 ± 0.01a	0.00 ± 0.00b
30	55 ± 0.01a	48 ± 0.01a	0.00 ± 0.00b
45	43 ± 0.05a	0.00 ± 0.00b	0.00 ± 0.00b
60	0.00 ± 0.00b	0.00 ± 0.00b	0.00 ± 0.00b

Treatments with the same letters are not statistically different.

### Metal accumulation profile in *Pleurotus *sp*.*

As shown in Table 4, with increasing heavy metal concentrations in liquid culture, the amount of heavy metal accumulation in the fungus mycelium also increases.

**Table 4:** Comparison of the average metal content (mg/gDw) of *Pleurotus* sp*.* mycelia grown in MMN medium containing heavy metals based on Duncan's test.

Metal concentration (mg/L)	Co	Cu	Ni
0	0.0 ± 0.00a	0.381 ± 0.13a	–
15	0.083 ± 0.001a	0.412 ± 0.07a	–
30	0.143 ± 0.005a	0.721 ± 0.1b	–
45	0.232 ± 0.005a	–	–
60	–	–	–

Treatments with the same letters are not statistically different.

### Antioxidative enzyme activity

Exposure of fungus to excess supply of each metals led to an increase in total SOD and CAT activity (Table 5).

**Table 5:** Comparison of the average enzymes activity (U/mg protein) of *Pleurotus* sp*.* mycelia grown in MMN medium containing heavy metals based on Duncan's test.

Metal concentration (mg/L)	Co		Cu	
	CAT activity (U/mg protein)	SOD activity (U/mg protein)	CAT activity (U/mg protein)	SOD activity (U/mg protein)
Control	0.753 ± 0.053b	3.82 ± 0.28c	3.541 ± 0.32b	1.3 ± 0.37c
15	1.427 ± 0.16b	4.04 ± 0.33c	4.012 ± 0.61b	1.81 ± 0.24c
30	2.96 ± 0.32a	4.86 ± 0.33bc	4.38 ± 0.26ab	4.5 ± 0.27a
45	3.85 ± 0.32a	7.13 ± 0.28a	5.44 ± 0.29a	5.34 ± 0.29a
60	3.88 ± 0.35a	4.93 ± 0.54b	5.79 ± 0.34a	3.89 ± 0.07b

Treatments with the same letters are not statistically different.

## Discussion

Mycoremediation is an approach that uses fungi that have a high ability to produce extracellular degrading enzymes. These fungi are used to remove or reduce toxic pollutants in water or soil. The threshold of the fungus in heavy metal tolerance is different depending on the type of species and the type and concentration of that heavy metal, so that inhibition of growth by a low concentration of one metal does not create an obstacle in the tolerance of another metal with a high concentration^23,24^. The variation in fungal response to trace metals is most likely due to intrinsic physiological factors. In the case of most fungal species, sensitivity to one or more types of metals does not mean sensitivity to all metals^23^. To evaluate the toxicity of heavy metals, *P. ostreatus* was exposed to three heavy metal cations, copper, cobalt, and nickel, and the growth rate of colony diameter in solid medium, dry weight of mycelium in liquid medium, and bioabsorption rate of metals were evaluated.

The investigating the stress effects of three elements cobalt, nickel and copper on mycelia growth *P.* sp*.* in liquid culture showed that the two elements cobalt and copper (< 30 mg/L) and cobalt (< 45 mg/L) did not notably suppress hyphal growth, while the high concentration of copper (> 30 mg/L) and cobalt (> 45 mg/L) significantly suppresses the growth of hyphae, Therefore, it shows that high concentrations of copper and cobalt were toxic to hyphae. Nickel inhibited growth even at the lowest concentration. Our study revealed that *P.* sp*.* is sensitive to Ni^2+^ cation in vitro. There is a negative relationship between fungus growth and heavy metal uptake. The reduction of the tolerance index in the case of nickel metal at the same initial concentrations and in the case of two metals cobalt and nickel at concentrations higher than 30 mg/L indicates the inhibitory role of these metals on the growth of fungus. This inhibition of growth by heavy metals may be due to Reactive Oxygen Species (ROS) from the Fenton reaction^25^.

El‐Sayed et al.^26^ reported that the inhibitory effect of iron metal on fungal growth *Aspergillus flavus* MT639638 appeared to be concentration-dependent, so that at the concentration of 1000 mg/L of iron, the mushroom growth was completely inhibited. Li et al.^27^ described a similar phenomenon and reported that the growth of the fungus *Pleurotus* sp. HAU-2 was inhibited by high concentrations of Cd (> 30 mg/L) and Cr (> 200 mg/L), also Chuang et al.^28^ reported the inhibition of fungal growth *Ganoderma lucidum* at 0.5 mM cadmium concentration.

There is a difference in the tolerance of the three metals copper, aluminum and zinc by *Laccaria laccata* species, so that the inhibition of fungal growth was observed at concentrations above 10 ppm of aluminum and copper^29^.

In the case of *Thelephora terrestris* species, high tolerance of this fungal species to high concentrations of copper (500 ppm) and zinc (1000 ppm) has been reported. The high tolerance of three fungal species *Hymenogaster* spp., *Scleroderma citrinum* and *Pisolithus tinctorius* in tolerating high concentrations of metals such as copper, iron, aluminum and zinc has also been reported^30^. Regarding the difference in metal tolerance in *Pleurotus sajor-caju*, which has the ability to grow in soils rich in metal elements, it has also been reported that this fungus has the ability to absorb cadmium and copper ions more than mercury and cobalt^31^. Biosorption of a wide range of metal pollutants, including heavy metals, by two fungal species *Pleurotus tuberregium* and *P.* sp*.* has also been reported^32^. The accumulation of heavy metals in the fruit body increases with the increase of metals in the substrate^33^. In the case of two different species of the *Pleurotus genus*, *P.* sp*.* and *P. flabellatus*, there is also a difference in the tolerance of the two metals cadmium and mercury^34^.

Examining the results showed a significant increase in the activity of antioxidant enzymes up to the concentration of 45 mg/L in the case of two metals, copper and cobalt, which is due to the antioxidant reaction of the fungus to detoxify heavy metals. El‐Sayed et al.^26^ reported that the enhanced synthesis of the antioxidative enzymes suggested that oxidative stress plays a major role in *Fusarium* *oxysporum* strain growing in the presence of Fe (II). An increase in the activity of antioxidant enzymes such as superoxide dismutase, catalase and lipid peroxidase has also been reported in the case of *Pleurotus florida* spp. in culture medium containing high amounts of various heavy metals^35^.

An increase in the activity of catalase and superoxide dismutase enzymes has also been reported in the case of *Trichosporon cutaneum R57*, which was exposed to high concentrations of chromium, copper and cadmium. Increased activity of intracellular enzyme system such as superoxide dismutase (SOD), metallothionein, reduced glutathione (GSH), lipid peroxidase and catalase in *Pleurotus* spp. play an important role in the intracellular accumulation of toxic metal ions inside the cell^36^.

## Conclusion

The outcome demonstrated that metal tolerance varied significantly, indicating that *P.* sp*.* was more tolerant of Co than other metals, so the fungus grew more in the presence of low concentrations of Co and Cu. However, even at concentrations as low as 15 mg/L, Ni greatly inhibited the growth of biomass and colony diameter. Heavy metals induced catalase (CAT) activity in superoxide dismutases (SOD) up to 45 mg/L, but an increase in metal concentration above 45 mg/L resulted in a significant decrease in SOD activities. Metals in mycelium also increased as the concentrations of these heavy metals increased.

## Data Availability

All data generated or analyzed during this study are included in this published article.
